# Synergistic Interaction Between *MALAT1/miR‐30b‐5p/BAFF* Axis and Inflammatory Cytokines Underlies Rituximab‐Refractory NMOSD

**DOI:** 10.1002/cns.70973

**Published:** 2026-06-08

**Authors:** Meiqun Deng, Keyi Zeng, Wei Chen, Jing Chen, Xiaoting Lin, Lei Chen, Yuxin Yao, Hanfei Chen, Aiyu Lin

**Affiliations:** ^1^ Department of Neurology, The First Affiliated Hospital Fujian Medical University Fuzhou China; ^2^ Fujian Institute of Neurology, The First Affiliated Hospital Fujian Medical University Fuzhou China; ^3^ The First Clinical College of Fujian Medical University Fuzhou Fujian China; ^4^ Department of Neurology, National Regional Medical Center, Binhai Campus of the First Affiliated Hospital Fujian Medical University Fuzhou China; ^5^ Department of Neurology Zhangzhou Affiliated Hospital of Fujian Medical University Zhangzhou Fujian China

**Keywords:** *MALAT1*/miR‐30b‐5p/BAFF, monocyte, neuromyelitis optica spectrum disorder, rituximab‐refractory

## Abstract

**Background and Objective:**

Despite achieving complete peripheral B cell deletion after over 6 months of rituximab therapy, a clinically significant minority of neuromyelitis optica spectrum disorder (NMOSD) patients experience breakthrough relapses, yet the underlying mechanism remains unclear. Our study aims to explore the underlying mechanism—the contribution of the *MALAT1*/miR‐30b‐5p/BAFF axis activation in monocytes to NMOSD disease attacks and relapses resistant to rituximab therapy.

**Methods:**

To investigate immune dysregulation in NMOSD, we established clinical groups comprising peripheral blood mononuclear cells (PBMCs) and serum from NMOSD patients: onset cases (PBMCs: *n* = 10; serum: *n* = 23) and rituximab‐treated cases subdivided into remission (*n* = 26) and rituximab‐refractory relapse (*n* = 13), along with healthy controls (*n* = 15). To profile the *MALAT1*/miR‐30b‐5p/BAFF axis and inflammatory mediators, we quantified *MALAT1*, miR‐30b‐5p, BAFF, and cytokine levels using qPCR, ELISA, and Western Blot and validated molecular interactions via dual‐luciferase reporter assays. To characterize cellular heterogeneity, single‐cell RNA sequencing (scRNA‐seq) was performed on PBMCs from two rituximab‐refractory relapsed NMOSD patients, two rituximab‐remission NMOSD patients, and two healthy controls. For mechanistic validation, THP‐1 cells were transfected with *MALAT1* overexpression constructs or miR‐30b‐5p mimics, followed by functional assessments to determine their roles in monocyte activation and cytokine production.

**Results:**

Significant activation of the *MALAT1*/miR‐30b‐5p/BAFF axis was observed in NMOSD patients at disease onset compared to healthy controls. Moreover, this axis correlated with rituximab‐refractory NMOSD, featuring monocyte‐specific *MALAT1* upregulation and elevated serum BAFF in rituximab‐refractory relapse patients. In THP‐1 cells, the overexpression of *MALAT1* downregulated miR‐30b‐5p, resulting in increased BAFF expression and pro‐inflammatory cytokines secretion (IL‐1β, IL‐8, TNF‐α), while also accelerating cell proliferation. Significantly, transfection with miR‐30b‐5p mimics reversed the *MALAT1*‐driven upregulation of BAFF and pro‐inflammatory cytokines, supporting the *MALAT1*/miR‐30b‐5p/BAFF axis as a regulatory circuit underlying inflammatory dysregulation in NMOSD.

**Conclusion:**

We identify the *MALAT1*/miR‐30b‐5p axis as a driver of NMOSD pathogenesis and rituximab‐refractory relapse. Upregulation of monocytic *MALAT1* suppresses miR‐30b‐5p, leading to increased BAFF and pro‐inflammatory cytokines secretion, thereby promoting inflammatory responses. Targeting this pathway may offer therapeutic potential for relapse prevention.

## Graphical abstract

The *MALAT1*/miR‐30b‐5p/BAFF axis is activated in monocytes during NMOSD onset and relapse. The *MALAT1*/miR‐30b‐5p/BAFF axis is activated in monocytes, thereby facilitating the release of BAFF and inflammatory cytokines (including IL‐1, IL‐6, IL‐8, and TNF‐α). These inflammatory cytokines recruit neutrophils and macrophages to infiltrate the central nervous system via a compromised blood–brain barrier, thus promoting the development of clinical attacks.

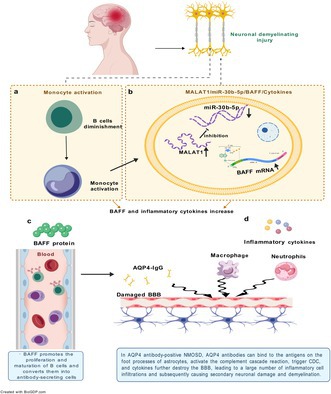

## 1 Introduction

Neuromyelitis optica spectrum disorder (NMOSD) is a severe autoimmune disorder affecting the central nervous system (CNS). It is characterized by the presence of pathogenic aquaporin‐4 (AQP4)‐IgG autoantibodies, which trigger recurrent optic neuritis and myelitis [1]. B cells play an essential role in the pathogenesis of NMOSD and are targeted by rituximab, a first‐line therapy that targets CD20‐positive B cells [2]. Rituximab has shown significant efficacy in treating NMOSD, leading to reductions in annual relapse rates and improvements in Expanded Disability Status Scale (EDSS) scores through the depletion of B cells and memory B cells [3]. However, rituximab‐refractory relapse remains a major clinical challenge [4], characterized by the occurrence of breakthrough disease activity despite effective depletion of peripheral B cells after over 6 months of rituximab therapy [5].

Current understanding attributes rituximab‐refractory NMOSD mainly to B cell‐related escape strategies and functional alterations, including B cells persistence in meningeal ectopic lymphoid tissue (mELT) [6], CXCR3‐mediated migration of B cells into the CNS [7], the survival of CD20‐negative plasma cells [8, 9], and the reconstitution from pre‐existing memory B cell reservoirs [10, 11]. However, increasing evidence suggests that non‐B cell‐mediated pathways also significantly contribute to disease progression. These pathways involve the activation of T, NK, NKT cells [12], monocytes, and dysregulation of the complement system [13]. Notably, in NMOSD, the non‐classical monocyte subset (CD14^+^CD16^++^) shows an increased frequency and enhanced IL‐6 production [14]. This not only implicates monocytes in the inflammatory pathogenesis, but also highlights the incomplete understanding of the underlying mechanisms.

Metastasis‐associated lung adenocarcinoma transcript 1 (*MALAT1*), a long non‐coding RNA (lncRNA), has been implicated in immune regulation by modulating the production of inflammatory mediators [15]. Studies have revealed that *MALAT1* disrupts myeloid homeostasis, inhibits antiviral innate immunity via TDP43‐mediated type I interferon (IFN‐I) [16], and can drive microglial over‐activation and neuronal apoptosis through the miR‐124‐3p/SGK1/NF‐κB axis [17], thereby accelerating the progression of autoimmune diseases. MicroRNAs (miRNAs) interacting with lncRNAs are involved in diverse biological processes. Abnormal expression of miRNAs is associated with various diseases including inflammation, cancer, and neurodegenerative disorders. Among these, miR‐30b‐5p has been reported to be associated with multiple inflammatory diseases. It can inhibit the expression of several downstream inflammatory factors, such as IL‐1β, IL‐6, TNF‐α, and CCL2 [18, 19, 20], suggesting its potential regulatory role in the pathogenesis of NMOSD. B cell‐activating factor (BAFF) is crucial in autoimmune nervous system diseases. It promotes the survival, proliferation, and clonal expansion of B cells in both the CNS and peripheral nervous system (PNS), thus facilitating the development of these diseases [21]. Furthermore, Wang‐Renault et al. have demonstrated that hsa‐mir‐30b‐5p can regulate BAFF expression by directly targeting its mRNA 3′ untranslated region (3′UTR) [22]. We have also observed the activation of a novel *MALAT1*/miR‐30b‐5p/BAFF regulatory axis in monocytes of NMOSD patients compared to healthy controls [23].

Although *MALAT1*, miR‐30b‐5p, BAFF, and monocytes have been linked to immune processes individually, their integration into a unified pathogenic axis underlying NMOSD onset and rituximab‐refractory relapse remains uninvestigated. We hypothesize that the dysregulation of a *MALAT1*/miR‐30b‐5p/BAFF axis within monocytes represents a novel non‐B cell pathway mediating relapse after rituximab‐induced B cell depletion. To test this hypothesis, our study aims to clarify the potential role of this axis in NMOSD pathogenesis and recurrence through clinical observations and in vitro validation and ultimately identify potential therapeutic targets for refractory cases.

## 2 Methods

### 2.1 Study Design and Patient Enrollment

This study received approval from the Ethics Committee of the First Affiliated Hospital of Fujian Medical University. A total of 62 NMOSD patients were enrolled in the First Affiliated Hospital of Fujian Medical University from January 2024 to April 2025. Inclusion criteria were as follows: (1) diagnosis of NMOSD based on the 2015 NMOSD diagnostic criteria developed by the international Panel for NMO Diagnosis (IPND); (2) testing sero‐positive for AQP4 antibody (confirmed by cell‐based assay [CBA]). The following were the exclusion criteria for patients with NMOSD: (1) testing sero‐negative for AQP4 antibody (CBA); (2) myelin oligodendrocyte glycoprotein antibody‐associated disease (MOGAD) (CBA) or multiple sclerosis (MS). Ultimately, 23 NMOSD patients at onset and 39 patients treated with rituximab (26 maintaining remission and 13 experiencing rituximab‐refractory relapse) were enrolled in the study groups. Onset refers to the first episode of the disease, and all onset samples were collected from treatment‐naïve patients prior to any immunomodulatory therapy including corticosteroids, immunosuppressants, or rituximab. Rituximab‐remission was defined as remaining relapse‐free for at least 6 months following rituximab treatment. Rituximab‐refractory refers to relapses occurring despite CD19^+^ B cells < 1% and CD27^+^ memory B cells < 0.05% in PBMCs after more than 6 months of rituximab therapy. Additionally, 15 age‐matched healthy controls (HCs) with negative autoimmune antibody panels were enrolled. Written informed consents were obtained from all study participants before sample collection. Clinical demographics, including age, sex, disease duration, core clinical characteristics, and EDSS score are presented in Table 1.

**TABLE 1:** Demographics and clinical characteristics of the participants.

Characteristics	Onset	Rituximab‐refractory patients	Rituximab‐remission patients	Healthy controls
Age, mean ± SD year	34.8 ± 12.5	42.8 ± 13.2	41.2 ± 12.1	39.0 ± 11.3
Sex, female, *n* (%)	22 (95.7)	11 (84.6)	23 (88.5)	13 (86.7)
Disease duration, mean ± SD (years)	—	13.1 ± 7.5	9.9 ± 7.1	—
Core clinical characteristics, *n* (%)				
Optic neuritis	10 (43.5)	6 (46.2)	12 (46.2)	—
Acute myelitis	12 (52.2)	7 (53.8)	12 (46.2)	—
Area postrema syndrome	1 (4.3)	—	2 (7.7)	—
EDSS score (median [range])	3.5 (2.0–4.5)	4.0 (2.0–7.0)	3.0 (2.0–6.0)	—

### 2.2 Blood Samples Processing

Blood samples were collected in heparin tubes for a total of 5 mL. The collected blood was mixed with an equal volume of phosphate buffered saline (PBS) and carefully layered onto 7 mL of Lymphoprep density gradient medium (TBDscience, China). Following centrifugation at 700 *g* for 25 min (24°C) without brake application, the lymphocyte interface was harvested, then washed twice (10 min at 300 *g*, 4°C, without brake) to remove platelets. The isolated peripheral blood mononuclear cells (PBMCs) were quantified and assessed for viability through trypan blue exclusion. Typically, approximately 30 million cells were harvested from each donor. The cells were cryopreserved in two aliquots (20 million cells per vial) using a cryoprotective solution composed of 90% FBS (Vazyme) and 10% DMSO (Solarbio) and subsequently stored in liquid nitrogen for future applications. All PBMCs were cryopreserved within 8 h after blood collection. For experimental procedures, thawed PBMC samples were evaluated for cell count and viability using a Nucleocounter NC‐200 instrument (Chemotec, 3450 Allerod, Denmark), demonstrating viability rates between 85% and 96%.

### 2.3 RNA Isolation and qRT‐PCR

Total RNA was isolated from PBMCs using TRIzol Reagent (Thermo Fisher Scientific, Invitrogen 15596018). RNA concentration and purity were assessed by measuring the OD260/OD280 nm absorbance ratios. 10 μg of total RNA was used for first‐strand cDNA synthesis using the Hifair AdvanceFast One‐step RT‐gDNA Digestion SuperMix (Yeasen) following the manufacturer's protocol. For miRNA reverse transcription, an additional 10 μg of total RNA was reverse‐transcribed into cDNA using the same kit, supplemented with miRNA‐specific stem‐loop primers. Quantitative PCR (qPCR) was conducted with Hieff UNICON Universal Blue qPCR SYBR Green Master Mix (Yeasen), using primers against miR‐30b‐5p, *MALAT1*, and BAFF. Involves primers: miRNA‐specific stem‐loop primers: 5′‐CGC GAG CAC AGA ATT AAT ACG ACT CAC TAT ACG CGA GCT GAG T‐3′; *MALAT1*‐Forward: 5′‐AAA GCA AGG TCT CCC CAC AAG‐3′, *MALAT1*‐Reverse: 5′‐GGT CTG TGC TAG TCA AAA GGC A‐3′; miR‐30b‐5p‐Forward: 5′‐GCC TGT AAA CAT CCT ACA CTC AGC‐3′, miR‐30b‐5p‐Reverse: 5′‐CGC GAG CAC AGA ATT AAT ACG‐3′; BAFF‐Forward: 5′‐AAA GGG GAA GTG CCC TAG AAG‐3′, BAFF‐Reverse: 5′‐TTT GCA ATG CCA GCT GAA TAG‐3′; IL‐6‐Forward: 5′‐CAA GAG TAA CAT GTG TGA AAG CAG CA‐3′, IL‐6‐Reverse: 5′‐TCT GCA GGA ACT GGA TCA GGA CT‐3′; HPRT‐Forward: 5′‐TGA CAC TGG CAA AAC AAT GCA‐3′, HPRT‐Reverse: 5′‐GGT CCT TTT CAC CAG CAA GCT‐3′; GAPDH‐Forward: 5′‐CTG GGC TAC ACT GAG CAC C‐3′, GAPDH‐Reverse: 5′‐AAG TGG TCG TTG AGG GCA ATG‐3′; U6‐Forward: 5′‐CTC GCT TCG GCA GCA CA‐3′, U6‐Reverse: 5′‐AAC GCT TCA CGA ATT TGC GT‐3′. Quantitative real‐time PCR was performed in 20 μL reaction volumes using the Rotor‐Gene Q system (Qiagen) with thermal cycling parameters: initial denaturation at 95°C for 5 min, followed by 40 cycles of 95°C for 10 s and 60°C for 30 s. Gene expression quantification was performed using the $2^{-∆∆C_{T}}$ method, with mRNA levels normalized to GAPDH and miRNA levels to U6 as the internal normalization reference.

### 2.4 Dual‐Luciferase Reporter Assay

miRNAs regulate target gene expression primarily through binding to the 3′ untranslated region (3′UTR) of mRNA, thus the target gene's 3′UTR was cloned downstream of the luciferase reporter gene (Table S1). To quantitatively investigate the regulatory interaction between *MALAT1* and miR‐30b‐5p, we designed six experimental groups and complementary positive controls. Plasmid configurations included: (1) 3′UTR‐NC (3′UTR‐negative control) with miRNA‐NC (miRNA‐negative control); (2) 3′UTR‐NC with miRNA mimics (hsa‐miR‐30b‐5p; 41827‐3); (3) 3′UTR (*MALAT1*; 52848‐1) with miRNA‐NC; (4) 3′UTR with miRNA mimics; (5) 3′UTR‐M (Mutant *MALAT1* 3′UTR; 52847‐1) with miRNA‐NC; (6) 3′UTR‐M with miRNA mimics; positive controls included: (1) positive control 3′UTR with miRNA‐NC; (2) positive control 3′UTR with positive control miRNA mimics. To validate the interaction ability between miR‐30b‐5p and BAFF, we also designed six experimental groups and complementary positive controls. Plasmid configurations included: (1) 3′UTR‐NC (3′UTR‐negative control; CON081) with miRNA‐NC (miRNA‐negative control; CON468); (2) 3′UTR‐NC with miRNA mimics (hsa‐miR‐30b‐5p; 109171‐2); (3) 3′UTR (*TNFSF13B*; 109212‐2) with miRNA‐NC; (4) 3′UTR with miRNA mimics; (5) 3′UTR‐M (Mutant *TNFSF13B* 3′UTR; 109229‐2) with miRNA‐NC; (6) 3′UTR‐M with miRNA mimics; positive controls included: (1) positive control 3′UTR with miRNA‐NC; (2) positive control 3′UTR with positive control miRNA mimics. Plasmid construction: vector: GV272 (Genechem, Shanghai, China) for *TNFSF13B*, GV716 (Genechem, Shanghai, China) for miR‐30b‐5p, XbaI digestion (Genechem, Shanghai, China).

HEK293T cells were plated at 1 × 10^5^ cells/well in 24‐well plates and cultured in 5% CO_2_ at 37°C until reaching 60% confluency. One microgram plasmid with 2 μL X‐tremeGENE HP transfection reagent (ROCHE) were mixed in 100 μL Opti‐MEM (20 min incubation). The plasmid‐reagent mixtures were then applied to cells for 5–6 h. Transfection efficiency was verified 24–48 h post‐transfection by fluorescence microscopy using co‐transfected GFP controls. Luciferase activity was measured 48 h post‐transfection using the Dual‐Luciferase Reporter System (Promega), with firefly signals normalized to Renilla controls. All experimental conditions were implemented with rigorous replication strategies, including three technical replicates per condition. Data were expressed as mean ± SD and statistically analyzed using one‐way ANOVA with Tukey's multiple comparisons test in GraphPad Prism 10.3.1. A probability threshold of *p* < 0.05 was applied to determine significance, with asterisks denoting specific *p*‐values (*p < 0.05, **p < 0.01, ***p < 0.001, ****p < 0.0001) in graphical representations.

### 2.5 Single‐Cell RNA Sequencing

The BD Rhapsody single‐cell analysis system utilizes CytoSeq's proprietary microwell‐based technology to capture individual cells. After quality control, a single‐cell suspension is loaded into the BD Rhapsody Cartridge, where magnetic beads—pre‐coated with cell‐labeling barcodes and UMIs—are introduced to co‐encapsulate cells within the microwells. Upon cell‐bead pairing, lysis buffer is added to release cellular RNA, allowing hybridization with barcoded oligos on the beads for cell‐specific molecular tagging. The RNA‐captured beads are then collected, quality‐checked, and subjected to reverse transcription to generate barcoded cDNA libraries. The bioinformatic analysis of single‐cell RNA sequencing (scRNA‐seq) data was conducted through the following workflow: (1) gene quantification using BD WTA Rhapsody Analysis Pipeline, (2) quality control filtering (eliminating low‐quality cells and low‐abundance genes), (3) expression normalization, followed by (4) downstream analyses including cellular heterogeneity assessment (dimensionality reduction, clustering, and marker‐based cell type annotation) and differential gene expression profiling with functional pathway enrichment analysis.

The R package “Seurat” was employed to detect differentially expressed genes (DEGs) between control and NMOSD samples. Genes that satisfied the thresholds of *p*‐values < 0.05 and fold change (FC) ≥ 1.5 were considered statistically significantly upregulated.

### 2.6 Construction of *MALAT1* Overexpression Cell Lines

The lentiviral vectors targeting *MALAT1* were constructed using the GV644 (Genechem, Shanghai, China, Table S2). Recombinant plasmids were co‐transfected into HEK293T cells with pHelper 1.0 and pHelper 2.0 plasmids. Viral particles were harvested 48 h post‐transfection through collection, concentration, and purification of the unpurified cell supernatants.

Lentiviral transduction of suspension cells was performed using optimized protocols from Genechem, Shanghai, China's suspension cell‐specific viral manual. For experimental procedures, cells in logarithmic growth phase were divided into three treatment groups: (1) Normal control (Nor) group: THP‐1 cells without transfection; (2) Negative Control (NC) group: transfected with empty vector; *MALAT1* Overexpression (OE) group: transfected with *MALAT1* lentiviral vector. THP‐1 monocytes were cultured in RPMI‐1640 medium supplemented with 10% FBS at 37°C in 5% CO_2_. THP‐1 cells were seeded at a density of 2 × 10^5^ cells/well in 6‐well plates containing 2 mL of complete medium supplemented with HiTransB‐1 infection enhancer. Forty microliter lentiviral particles with 80 μL HiTransB‐1 reagent were added to the cell suspension. Transduction efficiency was quantitatively evaluated at 48 h post‐infection through fluorescence microscopy analysis of GFP expression, demonstrating a cell adhesion integration rate approaching 100%. Cells were collected 48 h after transfection, and the expression of *MALAT1* was detected to determine whether the overexpressed *MALAT1* cell lines were successfully constructed. Quantitative PCR method of miR‐30b‐5p, *MALAT1*, BAFF, and pro‐inflammatory cytokines (IL‐1β, IL‐8, TNF‐α) was the same as the method mentioned above.

### 2.7 Cytokine ELISA

Serum was collected from 37 NMOSD patients (19 at acute phase and 18 in rituximab‐remission) and 15 healthy controls, and the concentrations of BAFF (EK1102) cytokine were analyzed using Human BAFF/BLys/*TNFSF13B* ELISA Kit (Multi Sciences, Hangzhou, China), following the manufacturer's instructions. Serum samples were diluted in a 1:2 ratio with buffer. The cytokine concentrations were quantified by referring to the standard curve.

### 2.8 Western Blot

The above THP‐1 cells were lysed in ice‐cold RIPA buffer supplemented with complete protease inhibitor cocktail (Thermo Fisher Scientific). After 30‐min incubation on ice, cell lysates were centrifuged at 12,000 × *g* for 10 min at 4°C to remove cellular debris. Protein concentrations were determined using the Bradford Protein Assay (Thermo Fisher Scientific) with bovine serum albumin (BSA) as the standard. For immunoblotting, equal protein amounts (30 μg per lane) were separated through 10% SDS‐PAGE using Prestained Protein Marker X (10–180 kDa, Servicebio) as a molecular weight reference, followed by electrophoretic transfer to PVDF membranes (0.45 μm pore size; Millipore, USA) under standard wet transfer conditions (100 V, 90 min). Membranes were blocked with 5% non‐fat dry milk in Tris‐buffered saline containing 0.1% Tween‐20 (TBST) for 1 h at room temperature, then incubated overnight at 4°C with primary antibodies: BAFF (TNFSF13B) Rabbit pAb (YT5166; Immunoway) and α‐tubulin Rabbit pAb (YN5437; Immunoway) as a loading control. Following three 10‐min TBST washes, membranes were incubated with secondary antibodies for 1 h at room temperature. Protein bands were visualized using enhanced chemiluminescence substrate (Thermo Fisher Scientific) and quantitatively analyzed using Quantity One software (version 4.6.9; Bio‐Rad). All experiments included three biological replicates, with band intensities normalized against corresponding tubulin expression levels. Band densities were quantified using Image J software.

### 2.9 Statistical Analysis

Data were expressed as mean ± SD and statistically analyzed in GraphPad Prism 10.3.1. Unpaired two‐tailed Student's *t*‐test was used for comparisons between two groups and one‐way ANOVA with Tukey's multiple comparisons test was used for comparisons between more than two groups. For data with significant heterogeneous variances (Levene's test, *α* = 0.1), Kruskal‐Wallis *H* test was used as a non‐parametric alternative to ANOVA, followed by post hoc pairwise comparisons using Dunn's test with Bonferroni adjustment. A probability threshold of *p* < 0.05 was applied to determine significance, with asterisks denoting specific *p*‐values (*p < 0.05, **p < 0.01, ***p < 0.001, ****p < 0.0001; ^#^ *p* < 0.05, ^##^ *p* < 0.01, ^###^ *p* < 0.001, ^####^ *p* < 0.0001) in graphical representations.

## 3 Results

### 3.1 The *MALAT1* /miR‐30b‐5p/BAFF Axis Activated in NMOSD Onset

**FIGURE 1:**
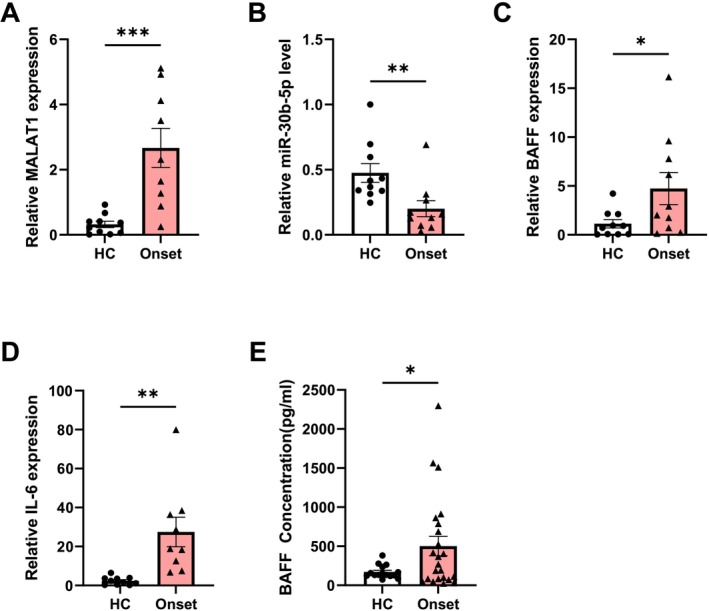
Altered expression of *MALAT1*, miR‐30b‐5p, BAFF and IL6 in NMOSD onset. (A–D) qRT‐PCR analysis measuring the expression of *MALAT1* (A), miR‐30b‐5p (B), BAFF and IL‐6 between NMOSD patients at onset and healthy controls. Relative *MALAT1* and IL6 expression: HC group: *N* = 10, onset group: *N* = 9. Relative miR‐30b‐5p level, relative BAFF expression: HC group: *N* = 10, NMOSD group: *N* = 10. (E) Cytokine ELISA assay measuring serum BAFF levels. HC, healthy control group, *n* = 15, NMOSD group: *N* = 23. *p < 0.05, **p < 0.01, ***p < 0.001, ns, no significant difference.

PBMCs were isolated from blood samples collected from 10 NMOSD patients during disease onset and 10 healthy controls. Total RNA was extracted from the PBMCs and subjected to qRT‐PCR analysis to measure the expression levels of *MALAT1* and miR‐30b‐5p. Analysis revealed that *MALAT1* expression was significantly upregulated in the onset group compared to the control group (*p* < 0.001; Figure 1A). Conversely, miR‐30b‐5p levels were significantly reduced in the onset group (*p* < 0.01; Figure 1B), demonstrating an inverse correlation with *MALAT1* upregulation. Next, we quantified the expression of BAFF and IL‐6—possible downstream targets of miR‐30b‐5p—in PBMCs using qPCR. Results indicated that BAFF and IL‐6 expression both increased in NMOSD patients at onset (Figure 1C,D). Additionally, serum samples from 23 NMOSD patients at onset and 15 healthy controls were analyzed. Serum BAFF levels, measured by ELISA, were significantly higher in the onset group compared to the HC group (*p* < 0.05; Figure 1E).

### 3.2 Validation of *MALAT1* /miR‐30b‐5p/BAFF Axis by Dual‐Luciferase Assay

**FIGURE 2:**
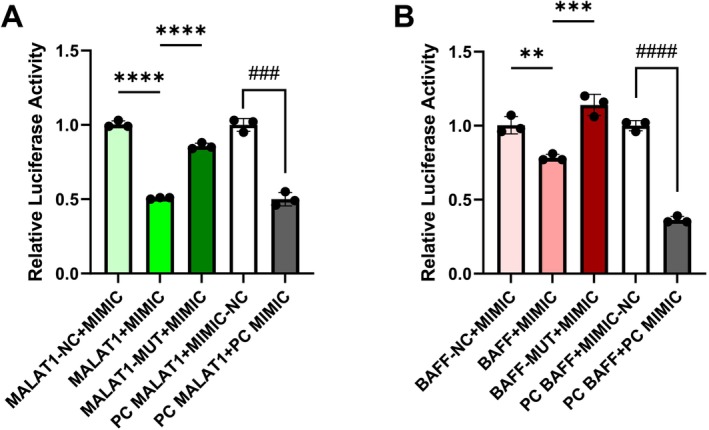
Validation of *MALAT1*/miR‐30b‐5p/BAFF regulatory axis by dual‐luciferase reporter assays. (A) Relative luciferase activity for dual luciferase activity reporter assay, positive control mimics reduced luciferase activity versus empty vector (*p* < 0.001), verifying proper transfection and detection. *MALAT1*, *MALAT1* 3′UTR; *MALAT1*‐NC, 3′UTR‐negative control; MIMIC, MiR‐30b‐5p mimics; MIMIC‐NC, MiRNA‐negative control; PC *MALAT1*, positive control *MALAT1* 3′UTR; PC MIMIC, positive control miR‐30b‐5p mimics. (B) Relative luciferase activity for dual luciferase activity reporter assay, positive control mimic reduced luciferase activity versus empty vector (*p* < 0.001), verifying proper transfection and detection. BAFF, *TNFSF13B* 3′UTR; BAFF‐NC, 3′UTR‐negative control; MIMIC, MiR‐30b‐5p mimics; MIMIC‐NC, MiRNA‐negative control; PC BAFF, positive control *TNFSF13B* 3′UTR; PC MIMIC, positive control miR‐30b‐5p mimics. **p < 0.01, ***p < 0.001, ****p < 0.0001; ^###^ *p* < 0.001, ^####^ *p* < 0.0001, ns, no significant difference.

To validate the regulatory relationship within the *MALAT1*/miR‐30b‐5p/BAFF axis, we performed dual‐luciferase reporter assays. These assays demonstrated that co‐transfection with a miR‐30b‐5p mimic significantly suppressed the luciferase activity of the wild‐type *MALAT1* plasmid (*p* < 0.0001; Figure 2A). In contrast, a mutant *MALAT1* plasmid with disrupted miR‐30b‐5p binding sites maintained luciferase activity similar to baseline levels, indicating specific binding between *MALAT1* and miR‐30b‐5p.

Similarly, dual‐luciferase reporter assays revealed that co‐transfection with the miR‐30b‐5p mimic significantly reduced the luciferase activity of the wild‐type BAFF 3′UTR plasmid compared to the BAFF negative control (*p* < 0.01; Figure 2B). Conversely, a mutant BAFF 3′UTR plasmid with altered binding sites exhibited significantly higher activity than the wild‐type plasmid when co‐transfected with the miR‐30b‐5p mimic (Figure 2B). These results confirm a direct binding interaction and functional regulation between miR‐30b‐5p and the BAFF 3′UTR.

### 3.3 Elevated Serum BAFF and Monocytic *MALAT1* in Rituximab‐Refractory NMOSD Relapse

**FIGURE 3:**
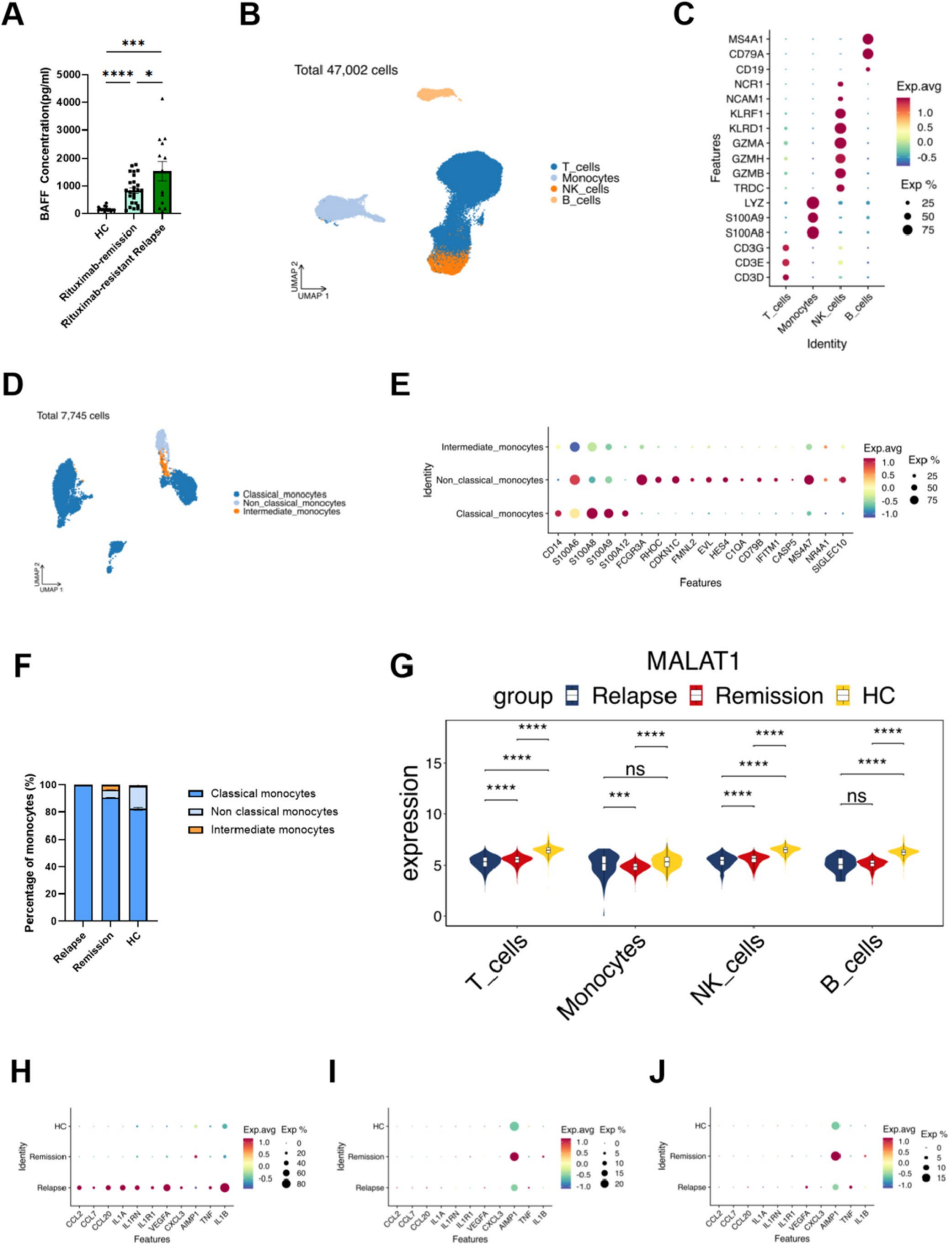
Alterations in BAFF, *MALAT1*, and pro‐inflammatory cytokines in rituximab‐refractory relapsing NMOSD patients. (A) Cytokine ELISA assay measuring serum BAFF levels in NMOSD rituximab‐refractory relapse group, rituximab‐remission group and healthy control group. HC, healthy control group, *n* = 15, rituximab‐remission group: *N* = 26, rituximab‐refractory relapse group: *N* = 13. (B–J) scRNA‐Seq analysis for PBMCs. (B) UMAP plot of PBMCs from all six samples (2 rituximab‐refractory relapsed NMOSD patients, 2 rituximab‐remission NMOSD patients, and 2 healthy controls), colored by four cell types (T, NK, B cells and monocytes). (C) Dot plot showing the identified four clusters with cell type‐specific markers. (D) UMAP plot colored by three cell types in monocytes (classical monocytes, non‐classical monocytes, and intermediate monocytes). (E) Dot plot showing the identified three monocyte subsets with cell type‐specific markers. (F) Proportions of classical, intermediate, and non‐classical monocyte subsets across groups. Each bar represents the mean proportion for each group (relapse, remission, and healthy control). Data are shown as mean ± SEM. (G) Violin plots show log‐normalized *MALAT1* expression levels across PBMCs in relapsed, remission, and healthy control samples (*n* = 2 per group). In monocytes, *MALAT1* was significantly elevated in relapsed patients compared to remission patients (***p < 0.001). In T cells and NK cells, *MALAT1* expression was lower in relapsed patients compared to remission patients. No significant differences were observed in B cells across groups (ns). (H–J) Dot plots showing the expression of pro‐inflammatory cytokines in monocytes (H), T cells (I), and NK cells (J) from relapsed patients (*n* = 2), remission patients (*n* = 2), and healthy controls (*n* = 2). *p < 0.05, ***p < 0.001, ****p < 0.0001.

During the analysis of serum BAFF levels in NMOSD patients, we made an unexpected observation: BAFF concentrations were significantly elevated in patients experiencing relapse compared to those in remission. This finding prompted us to further investigate BAFF in the context of rituximab‐refractory NMOSD. Despite being a first‐line NMOSD treatment through CD20^+^ B‐cell depletion, rituximab fails to prevent breakthrough relapses in a minority of patients. To validate the association between BAFF levels and rituximab‐refractory relapse, we enrolled a group of 39 NMOSD patients undergoing rituximab therapy, with 26 maintaining remission and 13 experiencing rituximab‐refractory relapse in the acute phase. Additionally, 15 healthy controls seronegative for disease‐relevant autoantibodies were included as a reference group. Serum BAFF levels were quantified by ELISA. Notably, the rituximab‐refractory relapse group exhibited significantly higher BAFF concentrations than both the rituximab‐sustained remission group (*p* < 0.001) and healthy controls (*p* < 0.001) (Figure 3A).

**FIGURE 4:**
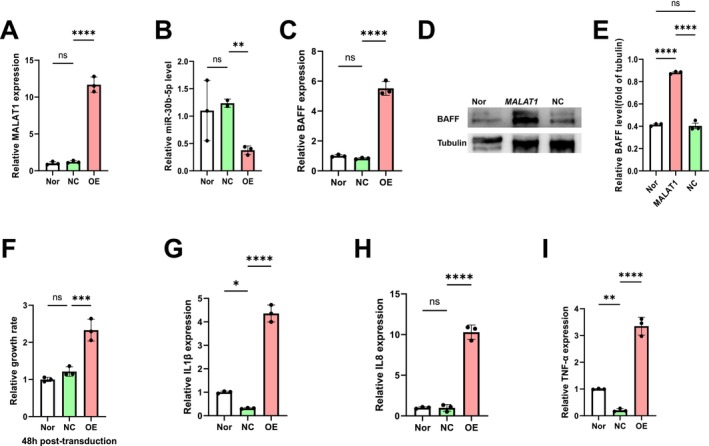
*MALAT1* overexpression in THP‐1 monocytes suppressed miR‐30b‐5p, elevated BAFF expression, and consequently facilitated cellular proliferation and inflammatory responses. (A) qRT‐PCR evaluation of *MALAT1* overexpression in THP‐1 cells. (B, C) qPCR analysis of miR‐30b‐5p and BAFF mRNA. NC, negative control group, THP‐1 cells transfected with empty vector; Nor, normal control group, THP‐1 cells without transfection; OE, *MALAT1* overexpression group, THP‐1 cells transfected with *MALAT1* lentiviral vector. (D, E) Western Blot detecting the expression levels of BAFF protein; the optical density values of protein bands were quantified and counted (normalized to tubulin), *MALAT1*: *MALAT1* overexpression group. (F) CCK‐8 determining THP‐1 cells' relative growth rate at 48 h post‐transduction. (G–I) qRT‐PCR analysis measuring expression levels of IL1, IL8, and TNF‐α in THP‐1 cells. *p < 0.05, **p < 0.01, ***p < 0.001, ****p < 0.0001, ns, no significant difference.

To identify which cell type(s) exhibit dysregulation of the *MALAT1*/miR‐30b‐5p/BAFF axis and may therefore contribute to relapse in rituximab‐refractory NMOSD patients, we performed single‐cell RNA sequencing (scRNA‐seq) on PBMCs from 2 rituximab‐refractory relapsed NMOSD patients, 2 rituximab‐remission NMOSD patients (stable disease with confirmed B‐cell depletion), and 2 healthy controls. Following rigorous quality‐control protocols, we acquired a total of 47,002 cells across all six samples. Uniform manifold approximation and projection (UMAP) dimensionality reduction segregated PBMCs into four transcriptionally distinct clusters: T cells, monocytes, NK cells, and B cells (Figure 3B,C). Given monocytes' recognized role as primary BAFF producers [24], we performed subclustering analysis on the monocyte compartment. Monocytes were divided into three subclasses: classical monocytes (CD14^+^, FCGR3A^−^), non‐classical monocytes (CD14^−^, FCGR3A^+^), and intermediate monocytes (CD14^−^, FCGR3A^−^, Figure 3D,E). Monocyte subset analysis revealed immune dysregulation in relapsed patients: the classical monocyte compartment was expanded, while the non‐classical monocyte compartment was reduced, compared to remission patients and healthy controls (Figure 3F). Differential expression gene (DEG) analysis revealed that *MALAT1* expression was significantly upregulated in monocytes from rituximab‐refractory relapsed patients compared to rituximab‐remission patients (Figure 3G). Interestingly, in T cells and NK cells, *MALAT1* expression was lower in relapsed patients compared to remission patients (Figure 4G). No significant differences in *MALAT1* expression were observed in B cells across the three groups (Figure 3G). Moreover, monocytes from rituximab‐refractory relapsed patients exhibited significantly increased expression of pro‐inflammatory cytokines (e.g., IL‐1B, TNF) compared to rituximab‐remission patients or healthy controls (Figure 3H). In contrast, no consistent upregulation of these cytokines was observed in T cells or NK cells (Figure 3I,J).

### 3.4 Overexpression of *MALAT1* Suppresses miR‐30b to Promote BAFF Production and Pro‐Inflammatory Factors in THP‐1 Cells

Single‐cell transcriptomics revealed a significant increase in *MALAT1* expression within monocytes of PBMCs from patients with rituximab‐refractory relapse. To investigate the functional consequences of this elevation, we overexpressed *MALAT1* in the monocytic cell line THP‐1 and assessed changes in downstream regulatory genes and their impact on cellular function. THP‐1 cells were transfected with a plasmid containing the *MALAT1* overexpression vector. Fluorescence microscopy confirmed high transfection efficiency (approaching 100%) at 48 h post‐transfection. Subsequent qPCR demonstrated a 9‐fold increase in *MALAT1* expression levels compared to both untransfected cells and cells transfected with an empty vector (Figure 4A). We next examined the *MALAT1*/miR‐30b‐5p/BAFF axis in *MALAT1*‐overexpressing cells using qPCR and western blotting (WB). Results showed that *MALAT1* overexpression significantly suppressed miR‐30b‐5p expression (*p* < 0.01 vs. vector control; Figure 4B). Concomitantly, the transcriptional level of BAFF, a target of miR‐30b‐5p, was markedly increased (*p* < 0.0001 vs. vector control; Figure 4C). Consistent with these findings, WB analysis confirmed elevated BAFF protein expression (Figure 4D,E).

To determine whether activation of the *MALAT1*/miR‐30b‐5p/BAFF axis alters cellular activity, we assessed the impact of *MALAT1* overexpression on cell proliferation using the CCK‐8 assay and quantified the expression of several pro‐inflammatory cytokines via qPCR. Results demonstrated that *MALAT1* overexpression significantly enhanced cell proliferation compared to the negative control group (*p* < 0.001; Figure 4F). Furthermore, qPCR analysis revealed that *MALAT1* overexpression markedly promoted the expression of pro‐inflammatory cytokines, including IL‐1β (*p* < 0.0001), IL‐8 (*p* < 0.0001), and TNF‐α (*p* < 0.0001) relative to the negative control (Figure 4G–I).

### 3.5 miR‐30b‐5p Mimics Inhibit Inflammation Induced by *MALAT1* Overexpression

**FIGURE 5:**
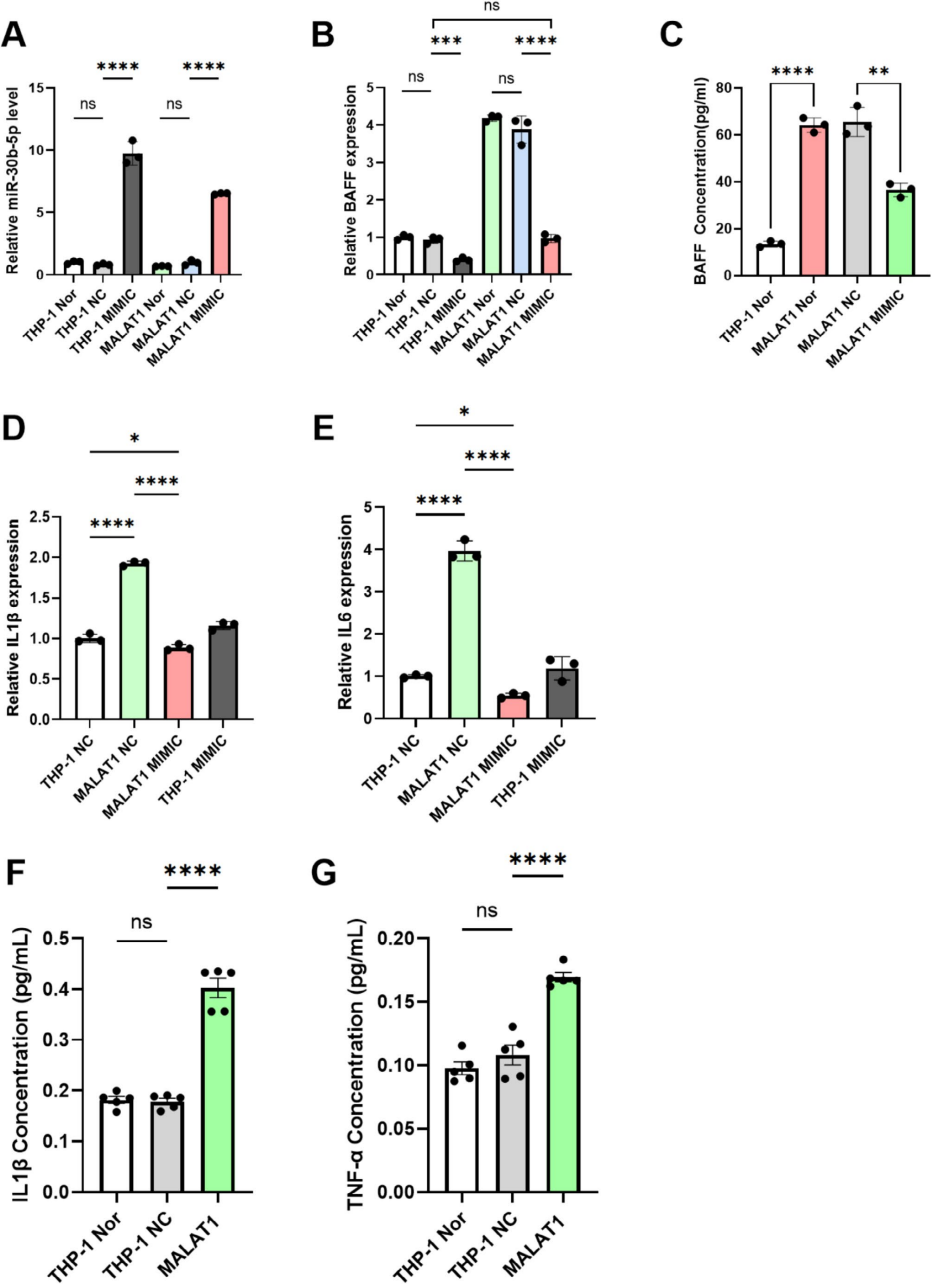
miR‐30b‐5p reconstitution reverses *MALAT1*‐driven BAFF, IL1β and IL6 upregulation. (A) Successful overexpression of miR‐30b‐5p was verified by qRT‐PCR (*p* < 0.0001 in THP‐1 cells, *p* < 0.0001 in *MALAT1*‐overexpressing THP‐1 cells). (B) qRT‐PCR analysis measuring expression levels of BAFF in THP‐1 cells. (C) Cytokine ELISA assay measuring BAFF concentration in THP‐1 cells. (D, E) qRT‐PCR analysis measuring expression levels of IL1β and IL6 in THP‐1 cells. (F, G) ELISA analysis of cytokine secretion in *MALAT1*‐overexpressing THP‐1 cells. *MALAT1* NC, *MALAT1*‐overexpressing THP‐1 cells transfected with empty vector; *MALAT1* Nor, *MALAT1*‐overexpressing THP‐1 cells without transfection; THP‐1 MIMIC, *MALAT1*‐overexpressing THP‐1 cells transfected with miR‐30b‐5p mimics; THP‐1 MIMIC, THP‐1 cells transfected with miR‐30b‐5p mimics; THP‐1 NC, negative control group, THP‐1 cells transfected with empty vector; THP‐1 Nor, normal control group, THP‐1 cells without transfection. *MALAT1*: *MALAT1* overexpression group. *p < 0.05, **p < 0.01, ***p < 0.001, ****p < 0.0001, ns, no significant difference.

Previous results indicated that activation of the *MALAT1*/miR‐30b‐5p/BAFF axis leads to decreased miR‐30b‐5p expression and increased proinflammatory cytokine production. To investigate whether restoring miR‐30b‐5p could mitigate this inflammation, we transfected miR‐30b‐5p mimics into *MALAT1*‐overexpressing cells and assessed downstream proinflammatory cytokine expression using qPCR and ELISA. First, qPCR analysis 48 h post‐transfection confirmed an eightfold increase in miR‐30b‐5p levels in cells transfected with the mimics (Figure 5A). Subsequent qPCR analysis of BAFF expression revealed that miR‐30b‐5p transfection significantly inhibited BAFF levels, irrespective of whether the cells were normal THP‐1 cells or *MALAT1*‐overexpressing cells (Figure 5B). This suppression of BAFF was further validated by ELISA detection of BAFF in the cell culture supernatant (Figure 5C). Finally, since *MALAT1* overexpression elevates not only BAFF but also other proinflammatory factors (e.g., IL‐1β and IL‐6), we measured these cytokines by qPCR. The results demonstrated that miR‐30b‐5p transfection significantly inhibited the *MALAT1* overexpression‐induced increases in IL‐1β and IL‐6 expression (Figure 5D,E). To further confirm that the elevated mRNA levels translated into increased protein secretion, we performed ELISA on the culture supernatant of *MALAT1*‐overexpressing THP‐1 cells. Consistent with the mRNA findings, ELISA analysis revealed significantly increased secretion of IL‐1β and TNF‐α in *MALAT1*‐overexpressing cells compared to control cells (Figure 5F,G).

### 3.6 Plasma From NMOSD Patients Upregulated *MALAT1* Expression in THP‐1 Cells

**FIGURE 6:**
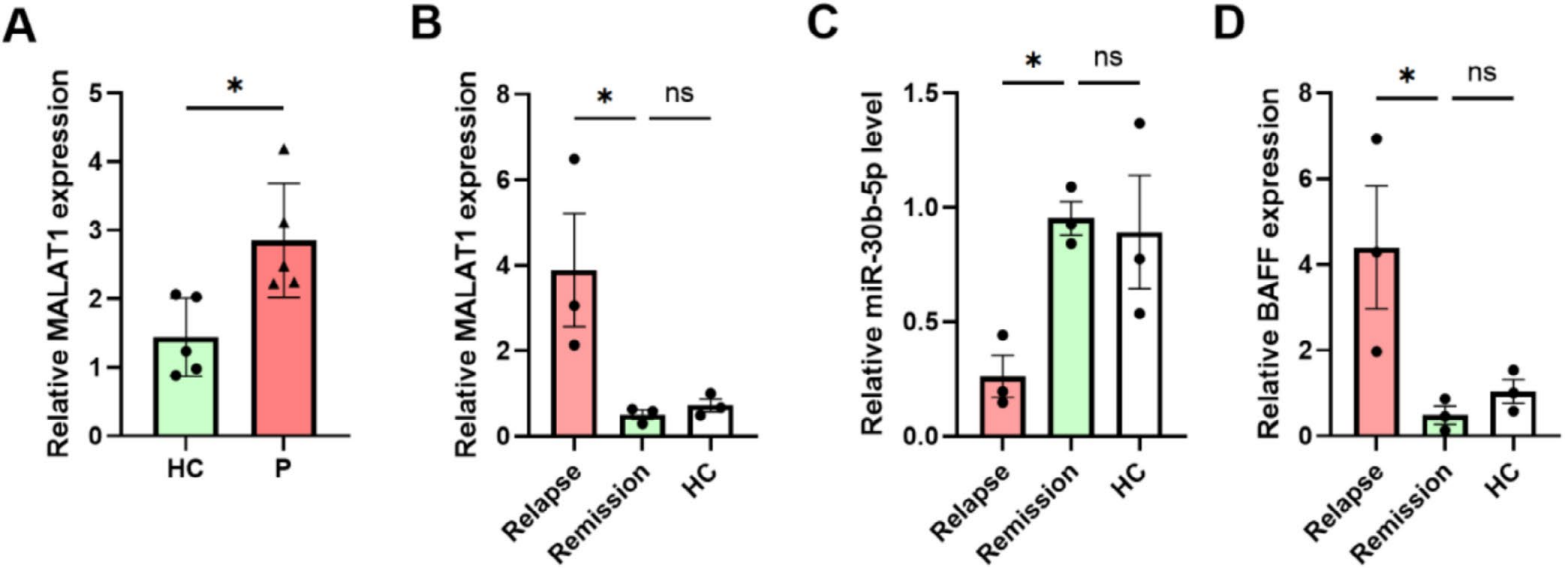
Plasma from NMOSD patients modulates *MALAT1* and BAFF expression in monocytes. (A) qRT‐PCR analysis of *MALAT1* expression in THP‐1 cells incubated with plasma from healthy controls (HC) or from NMOSD patients during acute relapse (P). (B–D) Primary monocytes isolated from healthy controls were treated with plasma from healthy controls (HC), NMOSD patients in remission (Remission), or NMOSD patients during acute relapse (Relapse) for 6 h. qRT‐PCR was performed to assess the expression levels of *MALAT1* (B), miR‐30b‐5p (C) and BAFF (D). HC, THP‐1 cells incubated with plasma of healthy controls; P, THP‐1 cells incubated with plasma of NMOSD patients during acute relapse. *p < 0.05.

The preceding findings indicate that elevated *MALAT1* levels in monocytes during NMOSD onset and recurrence suppress miR‐30b‐5p expression, leading to increased production of downstream pro‐inflammatory factors and thereby exacerbating disease progression and relapse. To preliminarily investigate the factors driving *MALAT1* elevation, we cultured THP‐1 cells with plasma from 5 active‐phase NMOSD patients and 5 age‐/sex‐matched healthy donors for 6 h. *MALAT1* expression was then quantified by qPCR and normalized to GAPDH. THP‐1 cells incubated with NMOSD patient plasma exhibited significantly higher *MALAT1* expression compared to those cultured with healthy donor plasma (*p* = 0.014, Figure 6A). These results suggest that factors present in the peripheral blood of NMOSD patients activate the *MALAT1*/miR‐30b‐5p/BAFF axis in monocytes, triggering the expression of downstream pro‐inflammatory factors and contributing to disease exacerbation.

To further validate these findings in a more physiologically relevant model, primary monocytes isolated from healthy donors were treated with plasma from three groups: healthy controls, NMOSD patients in remission, and NMOSD patients during acute relapse. After 6 h of incubation, qRT‐PCR analysis revealed that plasma from relapsed NMOSD patients significantly increased the expression of *MALAT1* (Figure 6B) and BAFF (Figure 6D) and significantly decreased the expression of miR‐30b‐5p (Figure 6C) in healthy donor‐derived monocytes compared with plasma from healthy controls or patients in remission. No significant differences were observed between the healthy control group and the remission group.

Collectively, these results demonstrate that circulating factors present in the plasma of relapsed NMOSD patients drive the upregulation of *MALAT1* and BAFF, as well as the downregulation of miR‐30b‐5p in monocytes, supporting the involvement of the *MALAT1*/miR‐30b‐5p/BAFF axis in disease activity and its potential contribution to rituximab‐refractory NMOSD.

## 4 Discussion

Our study reveals a novel pathogenic axis centered on *MALAT1*/miR‐30b‐5p/BAFF that drives both disease onset and rituximab‐refractory relapse in NMOSD. Importantly, we demonstrate that dysregulated monocytes act as the cellular hub for this axis, linking transcriptional alterations to clinical treatment failure. At disease onset in NMOSD patients, PBMCs exhibited significant upregulation of the lncRNA *MALAT1* with suppression of miR‐30b‐5p. This synergistic relationship was mechanistically validated by dual‐luciferase assays, which confirmed that *MALAT1* directly binds to miR‐30b‐5p. This dysregulation led to increased downstream effectors BAFF and IL‐6 expression in PBMCs and serum, amplifying B cell activation and neuroinflammation. These findings extend previous work implicating BAFF in NMOSD pathogenesis [21, 25] by defining its regulation via the lncRNA‐miRNA ceRNA network, revealing *MALAT1* as a master upstream regulator.

The clinical impact of this axis is most pronounced in the context of rituximab‐refractory NMOSD. We observed markedly elevated BAFF levels—both in serum and PBMC transcripts—in patients experiencing relapse despite B cell depletion therapy. This suggests that monocyte‐derived BAFF, being unaffected by anti‐CD20 agents, sustains survival signals for residual B cell precursors, enabling reconstitution of autoreactive clones and breakthrough disease activity. A similar rituximab‐refractory mechanism has also been reported in other autoimmune disorders [8, 26, 27]. However, as we know, this mechanism has been reported for the first time in NMOSD. The correlation between BAFF elevation and acute relapse further nominates serum BAFF as a predictive biomarker for treatment failure, potentially guiding early transition to BAFF‐targeted therapies (e.g., belimumab [28, 29, 30, 31, 32] and telitacicept [33, 34, 35]).

Single‐cell transcriptomics analysis revealed that classical monocytes (CD14^+^FCGR3A^−^) are the dominant source of *MALAT1* dysregulation in rituximab‐refractory relapse. The expansion of this pro‐inflammatory subset and contraction of immunoregulatory non‐classical monocytes creates a conducive environment for BAFF overproduction. Functional validation in THP‐1 monocytes confirmed that *MALAT1* overexpression led to suppression of miR‐30b‐5p, resulting in increased BAFF transcription, enhanced pro‐inflammatory cytokine production (IL‐1β, IL‐8, TNF‐α), and accelerated cellular proliferation. Crucially, this cascade is reversible: transfection with miR‐30b‐5p mimics normalized BAFF and cytokine levels, demonstrating the axis's therapeutic tractability.

Notably, plasma from active NMOSD patients induced *MALAT1* upregulation in THP‐1 cells, implicating circulating factors (e.g., autoantibodies, cytokines, or damage‐associated molecules) in initiating this pathogenic loop. Although the precise triggers remain to be identified, these findings suggest a self‐amplifying cycle wherein initial inflammation releases plasma mediators that activate monocytic *MALAT1*, further escalating BAFF and cytokine production to fuel relapse. This finding aligns with observations by Lehmann‐Horn et al. [36], who reported that the anti‐CD20 B cell depletion therapy in NMOSD caused hyperactivation and expansion of pro‐inflammatory monocytes.

Despite these advances, limitations warrant consideration. Although our scRNA‐seq analysis revealed elevated *MALAT1* expression specifically in monocytes, the small sample size necessitates validation in larger cohorts to confirm these findings. Moreover, longitudinal studies tracking BAFF dynamics post‐rituximab are necessary to establish its utility as a reliable biomarker. Future critical steps include identifying the plasma factors that induce *MALAT1* upregulation—a phenomenon observed in our plasma‐THP‐1 coculture experiments (Figure 6). Therapeutically, in vivo evaluation of *MALAT1*‐targeting antisense oligonucleotides [37, 38] and miR‐30b‐5p agomirs [39] in NMOSD models is warranted, particularly for rituximab‐refractory cases where our data indicate pathway reactivation. Additionally, exploring the mechanistic crosstalk between this axis and complement activation in AQP4‐IgG+ subgroups is essential, as synergistic effects may exacerbate injury. In conclusion, we propose that monocyte‐intrinsic *MALAT1*/miR‐30b‐5p/BAFF dysregulation is a pivotal mechanism underlying the progression of and treatment response in rituximab‐refractory NMOSD. Last but not least, direct qPCR validation using sorted patient monocytes was not performed due to sample exhaustion and the difficulty of re‐recruiting rituximab‐refractory NMOSD patients. Future studies with larger cohorts should validate monocyte‐specific *MALAT1* upregulation using purified CD14+ monocytes. Targeting this axis—through *MALAT1* silencing, miR‐30b‐5p restoration, or BAFF blockade—may offer novel strategies to mitigate relapse in refractory NMOSD.

## 5 Conclusion

This study identifies the *MALAT1*/miR‐30b‐5p axis as a driver of NMOSD pathogenesis and rituximab‐refractory relapse. We demonstrate that plasma mediators induce monocytic *MALAT1*, repressing miR‐30b‐5p and amplifying BAFF/pro‐inflammatory cytokines, thereby fueling inflammatory attack and relapse. Targeting this pathway may offer promising novel therapeutic strategies to prevent relapse in NMOSD.

## Author Contributions

Meiqun Deng was contributed to writing – original draft, conceptualization formal analysis and software. Keyi Zeng majored in formal analysis, software and methodology. Wei Chen participated in formal analysis, methodology and software. Jing Chen mainly participated in formal analysis, software and supervision. Xianxing Zhang, Xiaoting Lin and Lei Chen were majored in investigation, methodology and software. Yuxin Yao and Hanfei Chen contributed to conceptualization, methodology and software. Aiyu Lin was responsible for the conceptualization, formal analysis, supervision, resources and writing – review editing. All authors read and approved the final manuscript.

## Ethics Statement

The research related to human use has been complied with all the relevant national regulations, institutional policies and in accordance with the tenets of the Helsinki Declaration, and has been approved by the Ethics Committee of the First Affiliated Hospital of Fujian Medical University (approval number: [2025]179). All patient data were anonymized and handled in accordance with institutional and international ethical standards to ensure confidentiality and data protection. The patients included in this analysis were derived from a registered clinical cohort (NCT04386018).

## Consent

Written informed consent was obtained from individual or guardian participants.

## Conflicts of Interest

The authors declare no conflicts of interest.

## Supporting information


**Table S1:** The sequences of MALAT1, MALAT1‐mut, has ‐mir‐30b‐5p, BAFF and BAFF‐mut for dual‐luciferase assay.
**Table S2:** The sequences of LV‐MALAT1‐RNAi (P24K0244).

## Data Availability

The data that support the findings of this study are available from the corresponding author upon reasonable request.
