# Supplementary material for: Synergistic Interaction Between MALAT1/miR‐30b‐5p/BAFF Axis and Inflammatory Cytokines Underlies Rituximab‐Refractory NMOSD
Source: CNS Neurosci Ther. 2026 Jun 8;32(6):e70973. doi: 10.1002/cns.70973 (PMC13244142; doi:10.1002/cns.70973)
Supplement: Supplementary file 1 — Table S1: The sequences of MALAT1, MALAT1‐mut, has ‐mir‐30b‐5p, BAFF and BAFF‐mut for dual‐luciferase assay. Table S2: The sequences of LV‐MALAT1‐RNAi (P24K0244). [file CNS-32-e70973-s001.docx]

Table S1: The sequences of MALAT1, MALAT1-mut, has -mir-30b-5p, BAFF and BAFF-mut for Dual-Luciferase assay

| NR_002819MALAT1-3UTR(hmiR30b-5p)-WT | AGCCCATCAATTTAATTTCTGGTGGTGCAGAAGTTAGAAGGTAAAGCTTGAGAAGATGAGGG**TGTTTAC**  GTAGACCAGAACCAATTTAGAAGAATACTTGAAGCTAGAAGGGGAAGTTGGTTAAAAATCACATCAAAAAGC TACTAAAAGGACTGGTGTTTCTTTTTCCCTTAGGTCTGTCTAGAATCCTAAAGGCAAATGACTCAAGGTGTAAC AGAAAACAAGAAAATCCAATATCAGGATAATCAGACCACCACAG**GTTTAC**  AGTTTATAGAAACTAGAGCAGTTCTCACGTTGAGGTCTGTGGAAGAGATGTCCATTGGAGAAATGGCTGGTA GTTACTCT |
| --- | --- |
| NR_002819MALAT1-3UTR(hmiR30b-5p)-MUT | AGCCCATCAATTTAATTTCTGGTGGTGCAGAAGTTAGAAGGTAAAGCTTGAGAAGATGAGGG**GTGGGCA** GTAGACCAGAACCAATTTAGAAGAATACTTGAAGCTAGAAGGGGAAGTTGGTTAAAAATCACATCAAAAAGC TACTAAAAGGACTGGTGTTTCTTTTTCCCTTAGGTCTGTCTAGAATCCTAAAGGCAAATGACTCAAGGTGTAAC AGAAAACAAGAAAATCCAATATCAGGATAATCAGACCACCACAG**TGGGCA**  AGTTTATAGAAACTAGAGCAGTTCTCACGTTGAGGTCTGTGGAAGAGATGTCCATTGGAGAAATGGCTGGTA GTTACTCT |
| hsa-mir-30b-5p | ACTCGAGCGGCCGCCACTGTGCTGGATATCTGCAGAATTCCACCACACTGGACTAGT**GGATCC**TTCAACAGAGTCTTACGTAAAGAACCGTACAAACTTAGTAAAGAGTTTAAGTCCTGCTTTAAACCAAGTTTCAGTTCATGTAAACATCCTACACTCAGCTGTAATACATGGATTGGCTGGGAGGTGGATGTTTACTTCAGCTGACTTGGAATGTCAACCAATTAACATTGATAAAAGATTTGGCAAGAATAGTATACAGAGGCTTGAA**ACCGGT**AATAAAATATCTTTATTTTCATTACATCTGTGTGTTGGTTTTTTGTGTGAATCGATAGTACTAACATACGCTCTCCATCAAAACAAAACGAAACAAAACAAACTAGCAAAATAGGCTGTCCCCAGTGCAAGTGCAGGTGCCAGAACATTTCTCTCTCGAGTCCATCGATACTAGTAAGGATCTGCGATCGCTCCGGTGCCCGTCAGTGGGCAGAGCGCACATCGCCCACAGTCCCCGAGAAGTTGGGGGGAGGGGTCGGCAATTGAACGGGTGCCTAGAGAAGGTGGCGCGGGGTAAACTGGGAAAGTGATGTCGTGTACTGGCTCCGCCTTTTTCCCGAGGGTGGGGGAGAACCGTATATAAGTGCAGTA |
| NM_006573.5TNFSF13B-3UTR(hmiR30b-5p)-WT | AGGGCGGAAAGATCGCCGTGTAAT**TCTAGA**AACACATAATCTCCAACAGAAGTTACTGAATACATTCATACTAATGTAATGTAATTTCCCTTTATTTCTTGCTCTTCTGTTTCAAACTGCTGCTATTGTAGTTTACATATCCCAACCTTTAAAAATATTCCTCTTATTAGCTTTATATTCACTTTATAGAAGTTGAGTTTTAATTAAAATTCTTGGCATCCTGAAGTATGTCACATTTTTCATTTTATAAAGTACCATACTTAAGAATGCTGTAATACTTATCTTTTATAACATGTTTCCTTCGCTTTGCTTGTCTTTTATGTCATCAGTTTTAACTGTTTACTTCATTTAACAGTTTACATCATTCAACAGTTTACTTCATTAAACAGTAGGTGGAAAAATAGATGCCAGTCTATGAAAATCTTCCCATCTATATCAAAATACTTTTCAAGGATATACT**TCTAGA**GTCGGGGCGGCCGGCCGCTTCGAG |
| NM_006573.5TNFSF13B-3UTR(hmiR30b-5p)-MUT | AGGGCGGAAAGATCGCCGTGTAAT**TCTAGA**AACACATAATCTCCAACAGAAGTTACTGAATACATTCATACTAATGTAATGTAATTTCCCTTTATTTCTTGCTCTTCTGTTTCAAACTGCTGCTATTGTATGGGCAATATCCCAACCTTTAAAAATATTCCTCTTATTAGCTTTATATTCACTTTATAGAAGTTGAGTTTTAATTAAAATTCTTGGCATCCTGAAGTATGTCACATTTTTCATTTTATAAAGTACCATACTTAAGAATGCTGTAATACTTATCTTTTATAACATGTTTCCTTCGCTTTGCTTGTCTTTTATGTCATCAGTTTTAACGTGGGCATTCATTTAACATGGGCAATCATTCAACAGTTTACTTCATTAAACAGTAGGTGGAAAAATAGATGCCAGTCTATGAAAATCTTCCCATCTATATCAAAATACTTTTCAAGGATATACT**TCTAGA**GTCGGGGCGGCCGGCCGCTTCGAG |

Table S2: The sequences of LV-MALAT1-RNAi(P24K0244)

| LV-MALAT1-RNAi(P24K0244) | NNTGNNNGGNTGTTAGAGAGATANTTGGAATTAATTTGACTGTAAACACAAAGATATTAGTACAAAATACGTGACGTAGAAAGTAATAATTTCTTGGGTAGTTTGCAGTTTTAAAATTATGTTTTAAAATGGACTATCATATGCTTACCGTAACTTGAAAGTATTTCGATTTCTTGGCTTTATATAT**CTTGTGGAAAGGACGAAACACCGGGGTCTTACTAGTCTAGAATTCCTCGAGGAATTCTAGACTAGTAAGACCTTTTTGAATTCTGCAGTAACGCCATT**TTGCAAGGCATGGAAAAATACCAAACCAAGAATAGAGAAGTTCAGATCAAGGGCGGGTACATGAAAATAGCTAACGTTGGGCCAAACAGGATATCTGCGGTGAGCAGTTTCGGCCCCGGCCCGGGGCCAAGAACAGATGGTCACCGCAGTTTCGGCCCCGGCCCGAGGCCAAGAACAGATGGTCCCCAGATATGGCCCAACCCTCAGCAGTTTCTTAAGACCCATCAGATGTTTCCAGGCTCCCCCAAGGACCTGAAATGACCCTGCGCCTTATTTGAATTAACCAATCAGCCTGCTTCTCGCTTCTGTTCGCGCGCTTCTGCTTCCCGAGCTCTATAAAAGAGCTCACAACCCCTCACTCGGCGCGCCAGTCCTCCGACAGACTGAGTCGCCCGGGTACCGCGATCGCCGCCACCATGGTGAGCAAGGGCGAGGAGCTGTTCACCGGGGTGGTGCCCATCCTGGTCGAGCTGGACGGCGACGTAAACGGCCACAAGTTCAGCGTGTCCGGCGAGGGCGAGGGCGATGCCACCTACGGCAAGCTGACCCTGAAGTTCATCTGCACCACCGGCAAGCTGCCCGTGCCCTGGCCCACCCTCGTGACCACCCTGACCTACGGCGTGCAGTGCTTCAGCCGCTACCCCGACCACATGAAGCAGCACGACTTCTTCAAGTCCGCCATGCCCGAAGGCTACGTCCAGGAGCGCACCATCTTCTTCANGACGACGGCACTACAGACCCGCGCCGAGGTGAGTNGAGGGCGAANACCCTNNNANGCATCGAGCTGAGGGCATCGACTCANNAGANGCNCATCNGGGNACAGCCTGNNGTACAACTACCANAGCNCACGTCTTANTATTCATGNNGAANAGCNANAN |
| --- | --- |
|  |  |
